# Sickness and sleep health predict frustration and affective responses to a frustrating trigger

**DOI:** 10.1038/s41598-020-80461-4

**Published:** 2021-01-15

**Authors:** Leonie J. T. Balter, Tina Sundelin, John Axelsson

**Affiliations:** 1 Stress Research Institute, Stockholm University, Stockholm, Sweden; 2 Department of Clinical Neuroscience, Karolinska Institutet, Solna, Sweden

**Keywords:** Signs and symptoms, Risk factors, Emotion, Human behaviour

## Abstract

Fluctuations in health and sleep are common, but we know surprisingly little about how these daily life stressors affect one's level of frustration and sensitivity to becoming frustrated. In this pre-registered study, 517 participants (*M*_age_ = 30.4, *SD* = 10.4) reported their current sickness symptoms, health status, sleepiness, and sleep duration and quality the previous night. They also rated their general frustration and mood before and after a mild frustration-eliciting task. In the task, participants were instructed to copy geometric shapes onto a piece of paper, without lifting the pen from the paper. Participants were given three minutes to copy the eight shapes, but in order to induce frustration half of them were unsolvable. The study was subsequently repeated in an independent sample (*N* = 113). Frustration increased in response to the task; however, those with the worst sickness symptoms or sleep health reduced or did not change their frustration levels. Instead, across both studies, frustration was already high at baseline for these individuals. These findings indicate that being sick or having poor sleep is related to high general frustration, but resilience to further frustration due to mild frustrating situations.

## Introduction

Common stressors, such as sickness (e.g., the common cold, inflammation) and poor sleep health (e.g., short sleep) have been associated with altered emotional states such as more irritable mood, less positive mood, and amplified negative emotional reactions under provoking conditions or in response to negative work events^1–6^. However, despite over a decade of research establishing the emotional consequences of sickness and poor sleep health, we know surprisingly little about how these stressors affect frustration.

People’s everyday life is profoundly emotional^7^. Emotions are an integral component of our daily lives that prompt different behaviours. For example, the experience of anger boosts the tendency to take action, and fear exaggerates the perception of risk^8^. Frustration is elicited when unresolved problems are encountered, such as in the presence of contextual or psychological barriers, which must be removed to fulfil goals, desires, or needs^9,10^. Frustration may in some cases function as a facilitator to solve and overcome a problem through its energising function^10^. However, it is more often experienced as a negative emotion, contributing to the prevalence of stressful social events^11^. Even a single frustrating situation can have deleterious outcomes, such as inducing destructive behaviour including vandalism and violent crime^12,13^. Furthermore, momentary frustration can negatively impact cognitive processes such as attention^14^. This has important implications for situations that are prone to frustration, for example in traffic, where faulty decisions can have major consequences.

One's needs cannot always be adequately satisfied and thus practically everyone will experience frustration from time to time. Daily life consists of numerous situations that can elicit frustration, e.g., queuing in traffic when late for work, being unable to communicate one's emotional needs to a partner, or simply the inability to open a jar of jam. However, some individuals tend to become more easily frustrated than others. It has been suggested that those with poor impulse control or lower emotional intelligence are more impacted by frustrating events^15–17^. Not only may there be differences between individuals, there are also indications that frustration is state-dependent (i.e., varying within a person). For example, one study has found that severe sleep deprivation, with 55 h of wakefulness, affects the interpersonal responses to hypothetical frustrating situations^17^. However, whether the sleep-deprived participants actually felt more frustrated remained untested. There have been reports of amplified neural and pupil responses to negatively arousing images with prolonged wakefulness (32–35 h), suggesting that sleep deprivation augments negative emotional responses^18–20^. However, partial and total sleep-deprivation studies have also reported decreased or no change in emotional reactivity to negative stimuli, as assessed through evaluation of emotional pictures or facial emotional expressiveness^21–24^. While one or two nights of total sleep loss may be rare outside the sleep laboratory, mild reductions in sleep duration and quality, and the presence of other stressors, such as symptoms of infection like a cough or a stuffy nose, or sickness-related depressed mood, occur on a day-to-day basis in many individuals. Even though small variations in sickness, inflammation, and sleep have each been shown to change social behaviour, impair emotional regulation and cognitive functioning, intensify stress, anxiety, and anger, and reduce overall wellbeing^3–5,25–29^—all of which can have direct implications for how we respond to our environment—we know very little of whether these common life stressors are associated with feeling frustrated and the sensitivity to becoming frustrated.

Therefore, the current pre-registered study addressed whether sleep health and mild sickness symptoms are associated with general frustration, as well as the sensitivity to becoming frustrated in the presence of a frustration trigger. Participants reported their sleep duration and quality during the previous night, and their current sleepiness and sickness symptoms. They then rated their general frustration and positive (i.e., optimism, focus, energy) and negative (i.e., depression, sadness, anxiety, anger) emotions before and after a mild frustration-eliciting task. In the task, participants were instructed to copy geometric shapes onto a piece of paper, without lifting the pen from the paper and without tracing over the same line twice. Participants were given 3 min to complete the eight shapes; but in order to induce frustration, half of them were unsolvable. Since it has been shown that a trigger is important for the manifestation of aggression, which can be instigated by frustration^30,31^, it was hypothesised that frustration at baseline, without a frustration trigger, would not be associated with sickness or sleep health. However, those with more sickness symptoms and poorer sleep health were expected to be more sensitive to becoming frustrated when engaged in a mild frustration-eliciting task.

## Results

### Participant characteristics

Participant characteristics are summarised in Table 1.

**Table 1:** Descriptive characteristics of the study population given as sample size (*n*) and percentage (%), and mean (*M*) and standard deviation (*SD*).

Variable	*n* (%)	*M* (*SD*)
*N*	517	
Age (years)		30.4 (10.4)
**Sex**		
Male	230 (46%)	
Female	265 (54%)	
**Depression-like sickness (range 0–105)**		41.2 (19.8)
Low	136 (26%)	18.3 (6.2)
Intermediate	255 (49%)	39.9 (7.5)
High	126 (24%)	68.7 (11.2)
**Infection-like sickness (range 0–83)**		12.3 (13.0)
Low	149 (29%)	1.1 (1.2)
Intermediate	248 (48%)	9.7 (4.0)
High	120 (23%)	31.5 (12.6)
**Self-rated health M (SD) (range 1–10)**		7.3 (1.8)
Low (poor) (< 6)	90 (17%)	4.2 (1.0)
Intermediate (6 or 7)	157 (30%)	6.7 (0.5)
High (good) (8, 9, or 10)	270 (52%)	8.6 (0.7)
**Sleepiness (range 1–9)**		4.6 (1.7)
Low (alert)	179 (35%)	2.7 (0.6)
Intermediate	195 (38%)	4.7 (0.4)
High (sleepy)	143 (28%)	6.7 (0.6)
**Sleep quality (range 1–5)**		3.5 (1.0)
Low (poor) (1 or 2)	97 (19%)	1.8 (0.4)
Intermediate (3)	127 (25%)	3.0 (0.0)
High (good) (4 or 5)	293 (57%)	4.2 (0.4)
**Sleep duration (range 1 h–13 h)**		7 h 48 min (1 h 30 min)
Short (< 7 h)	126 (24%) of which *n* = 55 < 6 h	5 h 52 min (1 h 02 min)
Normal (≥ 7 − < 9 h)	307 (59%)	8 h 00 min (0 h 33 min)
Long (≥ 9 h)	84 (16%)	9 h 48 min (0 h 38 min)

Percentages may not total 100 due to rounding.

### Task-induced frustration and emotional reactivity

Participants reported on average 1.3 attempts (*SD* = 2.0) for each solvable shape and 2.6 attempts (*SD* = 1.4) for each unsolvable shape. As shown in Table 2 and Supplementary Materials Fig. S1a, a significant task (baseline, task) effect indicated that frustration levels increased from baseline to during the task, showing that the frustration induction was successful. While frustration increased, anger, anxiety, depression, and sadness all decreased significantly (on average -11 points). Energy, focus, and optimism all increased (on average + 21 points) from baseline to during the task. Supplementary Materials Fig. S2 (sickness) and S3 (sleep health) show the statistics and mean ratings of negative and positive mood at baseline and during the Frustration Tolerance Task, separated by sickness and sleep health groups.

**Table 2:** Mean ratings (SEM) at baseline and during the Frustration Tolerance Task (rated immediately after the task, indicated as Task).

	Baseline	Task	*β*	95% CI	*p*
Frustration	34.9 (1.3)	43.9 (1.4)	0.29	0.18, 0.39	< 0.001
Anger	18.1 (1.1)	11.8 (0.9)	− 0.27	− 0.38, − 0.17	< 0.001
Anxiety	36.5 (1.3)	28.3 (1.3)	− 0.28	− 0.37, − 0.19	< 0.001
Depression	26.5 (1.2)	16.7 (1.0)	− 0.38	− 0.46, − 0.30	< 0.001
Sadness	29.8 (1.3)	11.0 (0.8)	− 0.72	− 0.81, − 0.63	< 0.001
Energy	31.1 (1.0)	44.3 (1.2)	0.50	0.41, 0.59	< 0.001
Focus	42.3 (1.2)	77.0 (0.9)	1.16	1.07, 1.25	< 0.001
Optimism	44.1 (1.1)	60.6 (1.2)	0.60	0.51, 0.70	< 0.001
Mental demand		66.6 (1.0)			
Physical demand		15.5 (0.9)			
Hurriedness		63.1 (1.3)			
Enjoyment		58.8 (1.2)			
Self-perceived performance		37.4 (1.0)			
Amount of effort used		72.8 (1.0)			
Feeling in control		51.0 (1.3)			
Motivation		73.0 (1.0)			
Excitement		51.4 (1.3)			
Shame		21.4 (1.3)			

Standardised coefficient estimates (β), 95% confidence intervals (CI), and p values are shown for task effects (baseline, task) for all mood variables. Higher scores (maximum of 100) indicate a greater level of the variable (e.g., higher anxiety, higher energy, greater effort used).

Replicating the study in an independent sample of 113 participants produced almost identical task responses (see Supplementary Materials Fig. S1b), indicating that the 10-point Likert scale can be validly rescaled to the 0–100 VAS and reliability of the results. All results of the replication study are shown in the Supplementary Materials 5.3.

### Sickness and sleep measures

**Figure 1:**
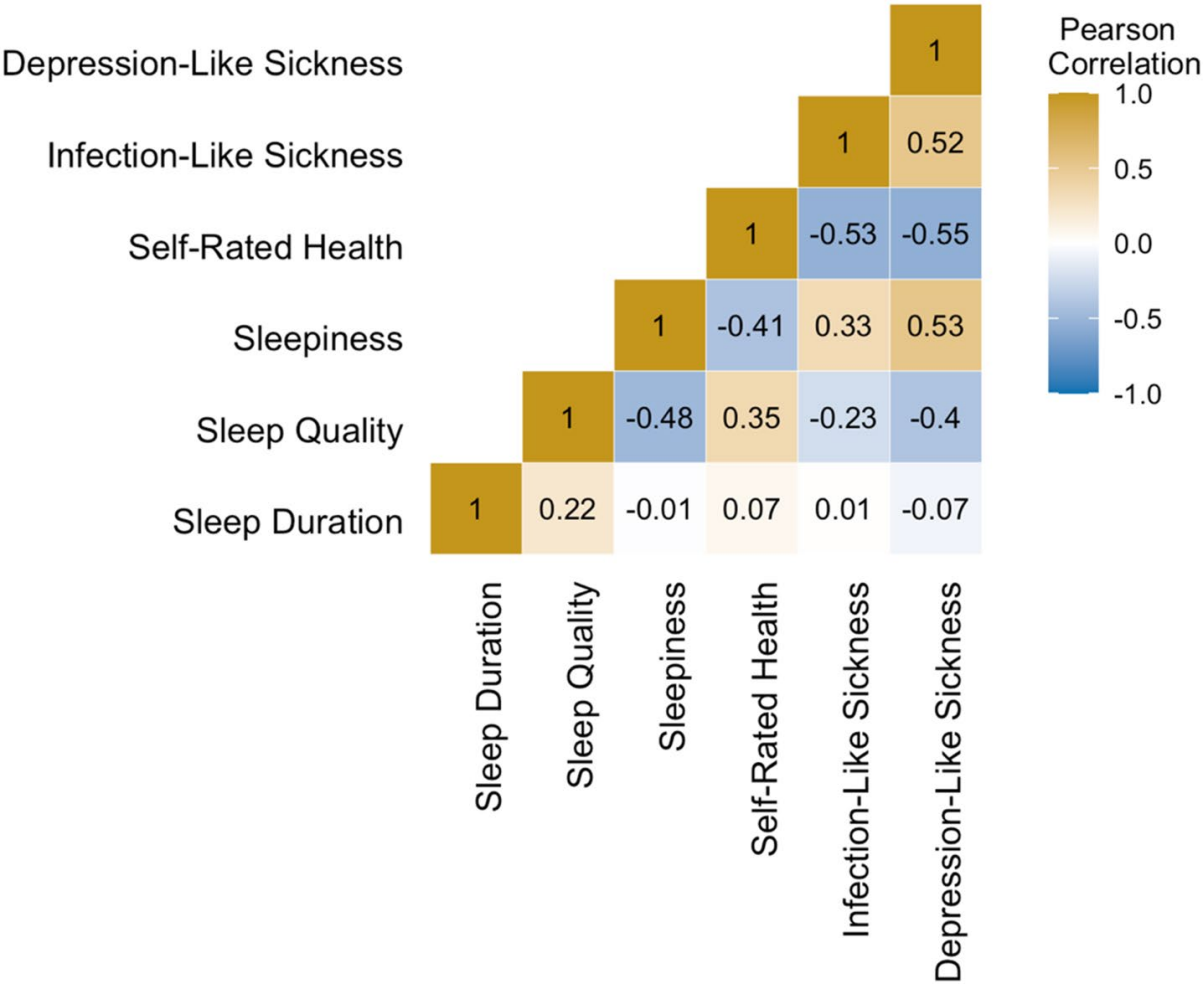
Heatmap showing Pearson correlation coefficients between all sickness variables (depression-like sickness, infection-like sickness, self-rated health) and sleep health variables (sleepiness, sleep quality, sleep duration).

The exploratory factor analysis extracted two factors, see Supplementary Material Table S1 for the factor loadings. Based on the symptoms that loaded onto each factor, Factor 1 (e.g., depressed, drained, do not wish to do anything) is referred to as ‘depression-like sickness’ and Factor 2 (e.g., cough, fever, muscle aches) as ‘infection-like sickness’. Figure 1 shows the Pearson correlation coefficients between all sickness and sleep health variables.

### Baseline frustration

#### Depression-like sickness, infection-like sickness, self-rated health

**Figure 2:**
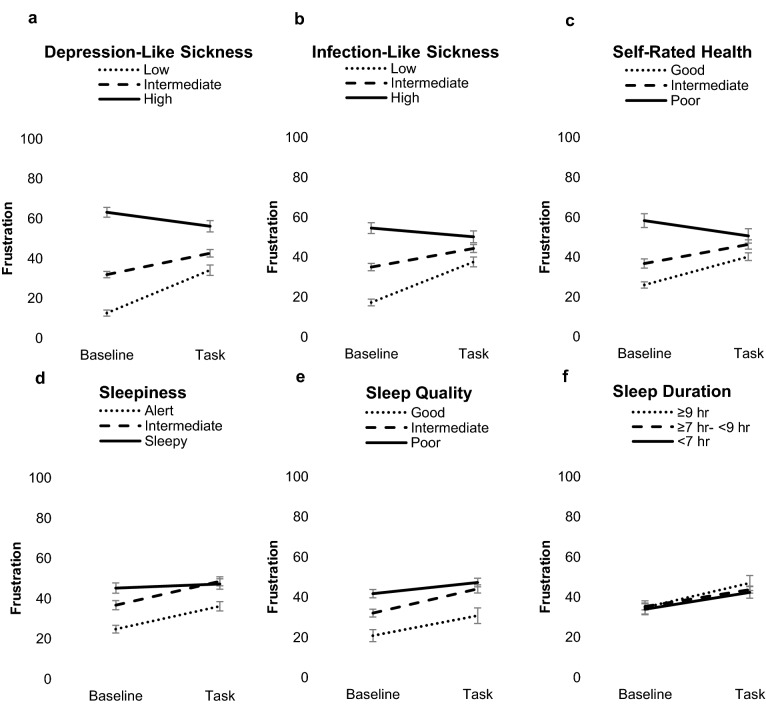
Frustration levels (means and SEM) at baseline and during the *Frustration Tolerance Task*, for the groups with low, intermediate, and high levels of sickness (**a**–**c**) and sleep health stressors (**d**–**f**) (the sleep duration groups was divided into short-, intermediate- and long sleepers).

All three sickness-related factors predicted frustration at baseline: depression-like sickness (*β* = 0.66, 95% CI [0.59, 0.72], *p* < 0.001), infection-like sickness (*β* = 0.49, 95% CI [0.41, 0.56], *p* < 0.001), and self-rated health (*β* = − 0.44, 95% CI [− 0.51, − 0.36], *p* < 0.001). As shown in Fig. 2 and Supplementary Materials Table S2 (original values before rescaling), those high in depression-like sickness and infection-like sickness, and those with low self-rated health reported higher frustration at baseline, followed by those moderate in these stressors. Low frustration was observed in those with low depression-like sickness, low infection-like sickness, and in those with high self-rated health (depression-like sickness: *F*(2, 517) = 148.59, *p* < 0.001; infection-like sickness: *F*(2, 517) = 69.41, *p* < 0.001; self-rated health: *F*(2, 517) = 45.36, *p* < 0.001).

#### Sleepiness, sleep quality, and sleep duration

Greater sleepiness (*β* = 0.28, 95% CI [0.20, 0.36], *p* < 0.001) and lower sleep quality (*β* = − 0.24, 95% [CI − 0.33, − 0.16], *p* < 0.001) were associated with higher frustration at baseline. As shown in Fig. 2 and Supplementary Materials Table S2 (original values before rescaling), frustration levels were highest in those with high sleepiness and in those with the worst sleep quality, followed by those with moderate sleepiness and sleep quality, and low frustration ratings were observed in those with low sleepiness and good sleep quality (sleepiness: *F*(2, 517) = 19.96, *p* < 0.001; sleep quality: *F*(2, 517) = 11.96, *p* < 0.001). Sleep duration did not significantly predict baseline frustration (*β* = 0.02, 95% CI [− 0.07, 0.10], *p* = 0.707) and frustration at baseline was not significantly different between short, normal, and long sleepers (*F*(2, 517) = 0.18, *p* = 0.836). There was neither a linear (*F* = 0.14, *p* = 0.707) nor quadratic (*F* = 1.34, *p* = 0.262) relationship evident between sleep duration and frustration.

### Task-induced frustration

#### Sickness and task-induced frustration

A task (baseline, task) × depression-like sickness interaction (*β* = − 0.33, 95% CI [− 0.43, − 0.23], *p* < 0.001) was evident for frustration. Similar effects were found for infection-like sickness (*β* = − 0.34, 95% CI [− 0.44, − 0.24], *p* < 0.001) and self-rated health (*β* = 0.28, 95% CI [0.18, 0.37], *p* < 0.001). As shown in Fig. 2 and Table 3, separating individuals into low, intermediate, and high level of each sickness type indicated that those with low or intermediate depression-like sickness or infection-like sickness, and those with high or intermediate self-rated health increased in frustration in response to the task. However, those with high depression-like sickness or low self-rated health decreased in frustration and those with high infection-like sickness did not significantly change (*F* = 3.71, *p* = 0.055) (task × depression-like sickness: *F*(2, 517) = 21.26, *p* < 0.001; task × infection-like sickness: *F*(2, 517) = 18.53, *p* < 0.001; task x self-reported health: *F*(2, 517) = 12.45, *p* < 0.001).

**Table 3:** Frustration levels (means and SEM) at baseline and during the frustration tolerance task, for the groups with low, intermediate, and high levels of sickness and sleep health stressors (the sleep duration groups was divided into short, normal, and long sleepers).

	Low/Short		Intermediate/Normal		High/Long	
	Baseline	Task	Baseline	Task	Baseline	Task
Depression-like sickness***	12.6 (0.9)	34.1 (1.9)***	32.5 (1.5)	42.6 (1.9)***	63.8 (2.7)	56.8 (2.6)***
Infection-like sickness***	17.2 (1.1)	37.6 (1.9)***	35.1 (1.7)	44.7 (2.0)***	56.2 (2.5)	49.9 (2.4)*
Self-rated health***	58.3 (2.1)	50.6 (2.0)*	36.7 (1.5)	46.3 (1.7)***	26.0 (2.1)	40.2 (2.8)***
Sleepiness*	24.7 (1.4)	36.1 (1.9)***	36.6 (1.9)	48.5 (2.2)***	45.1 (2.2)	47.2 (2.2)
Sleep quality (n.s.)	45.7 (2.3)	48.1 (2.4)	38.6 (2.0)	46.6 (2.3)	29.7 (1.5)	41.3 (1.8)
Sleep duration (n.s.)	33.5 (1.8)	43.5 (2.2)	35.4 (1.7)	43.2 (1.8)	34.8 (1.9)	46.9 (2.4)

Note that low depression-like sickness, low infection-like sickness, and low sleepiness indicate better health (i.e., less stress), while low sleep quality and low self-rated health indicate a worse level of the stressor. Short sleep duration refers to < 7 h, normal sleep duration refers to ≥ 7 to < 9 h, and long sleep duration to ≥ 9 h. n.s. = non-significant interaction between the stressor and task-induced frustration. *in the first column indicates significance level for interaction effects between the stressor and task effect (baseline, task). Other columns with * indicate significance level for post-hoc task comparisons (baseline, task) for each stressor level in case a significant interaction effect was evident, *p < .05, **p < .01, ***p < .001.

#### Sleep health and task-induced frustration

Task × sleep quality (*β* = 0.10, 95% CI [0.00, 0.20], *p* = 0.051) and task × sleepiness (*β* = − 0.11, 95% CI [− 0.21, − 0.01], *p* = 0.040) interactions were observed for frustration, although the former was borderline statistically significant. No significant task × sleep duration interaction was evident (*β* = 0.02, 95% CI [− 0.08, 0.12], *p* = 0.668). As shown in Fig. 2 and Table 3, separating individuals into low, intermediate, and high sleep health indicated that those high in sleepiness did not significantly change in frustration, while those with low or intermediate levels of sleepiness increased in frustration (sleepiness × task: *F*(1, 517) = 3.56, *p* = 0.029). The sleep duration x task (*F*(2, 517) = 0.53, *p* = 0.592) interaction was not statistically significant.

## Discussion

The current study examined how natural variation in sickness and sleep health were associated with frustration and the sensitivity to becoming frustrated. Contrary to our hypotheses, sickness (i.e., depression-like sickness, infection-like sickness, and low self-rated health) and worse sleep health (i.e., sleepiness, low sleep quality, but not sleep duration) were associated with greater frustration at baseline. The results further show that frustration increased in response to a frustration trigger, with larger increases for those with low or intermediate levels of sickness or sleep health stressors (except for sleep duration). Those high in these stressors decreased, or did not change their frustration levels in response to the frustrating situation, possibly because they were already showing high levels of frustration prior to engaging in the task. These results show that being sick or having poor sleep health is related to higher levels of frustration in general, but with a reduced reactivity when exposed to a mild frustrating situation.

While those with low or intermediate levels of sickness and those with high or intermediate sleep health were less tolerant to a frustration trigger, those with the worst sickness or sleep health decreased or did not change their frustration levels during the task. High frustration at baseline in those with worst sickness or sleep health may partly explain this apparent resilience to frustration induction. An alternative explanation might be that the task shifted their attention away from negative emotions caused by having worse sickness or sleep health. Indeed, exteroceptive cues have been found to bias interoceptive attention^32^. For example, engagement in a cognitive task can reduce subjective pain perception^33^. Engagement in the frustrating task may have resulted in attention away from interoceptive cues (e.g., experience of depressive feelings) towards external task cues (e.g., focus on drawing the shapes). In line with this explanation is the finding that negative mood decreased (-11 points on average) and positive mood, including focus, increased (+ 21 points on average) during the task. Indeed, those with worse sickness or sleep health showed 16 points larger decreases in negative mood and 5 points larger increases in positive mood as compared to those low in sickness or with high sleep health.

Previous work on sleep health, mood, and emotional reactivity in healthy individuals has focused mostly on the consequences of shortened sleep, and to a lesser extent on sleepiness and sleep quality. Short sleep and sleepiness are generally associated with impaired mood and compromised interpersonal functioning^2,3,34,35^. However, there is inconsistency regarding the direction of such sleep-related emotional reactivity; enhanced as well as reduced negative emotional reactivity has been reported using various types of outcome measures including neural and pupil responses, emotional ratings, and facial emotional expressiveness^19,21–24^. Moreover, most aforementioned studies assessed threat-related reactivity or general mood changes in response to negative and/or positive stimuli, leaving largely unexplored the role of sleep health in frustration reactivity. Berger et al.^36^ showed that sleep restriction in early childhood (i.e., nap deprivation) increased negative and decreased positive facial emotional responses in response to a frustrating task in the form of an unsolvable puzzle. Similarly, Krizan et al.^4^ suggested that experimentally reduced sleep duration in adults increases feelings of anger in response to a frustrating stimulus. However, importantly, subjective sleepiness mediated the relationship between sleep duration and feelings of anger, suggesting that sleepiness, rather than sleep duration per se, accounted for at least part of the anger response. The current study extends these findings, showing that sleepiness and low sleep quality, but not sleep duration, are associated with greater frustration in the absence of a trigger. The finding that feelings of sleepiness, low sleep quality, and sickness, but not self-reported sleep duration, were associated with frustration suggests that the subjective nature of reduced well-being may be particularly important for the experience of frustration. While Krizan et al.^4^ showed a role for sleepiness in intensified anger, the role of sleepiness in sensitivity to becoming angry could not be assessed since anger levels were not measured before provocation. Anger may thus have been elevated due to the sleep restriction, and related sleepiness, rather than the task. Indeed, it has been shown that daytime sleepiness is associated with greater anger, depression, anxiety, and intensity of psychological symptoms, even in the absence of a trigger^37^. Although we did find that frustration during a frustrating task was higher with greater sleepiness and lower sleep quality, it was already elevated at baseline. This finding highlights the importance of including baseline measurements when addressing emotional reactivity.

The majority of the participants became mildly frustrated by the task, without increasing other negative mood variables. Negative emotions and anger have often been found to increase during frustrating tasks^38,39^, but our results are in line with the updated frustration-aggression hypothesis of Berkowitz et al.^31^. Berkowitz et al.^31^ criticised the original frustration-aggression hypothesis of Dollard et al.^9^ which stated that the existence of frustration is always followed by anger and aggression. Instead, Berkowitz^31^ postulated that frustration can lead to aggressive inclinations, but only if connected with an increase in negative affect. Indeed, the Frustration Tolerance Task that was used in the current study induced frustration without increasing anger and other negative affect components, suggesting that the task can be utilised to specifically address frustration reactivity, separate from aggression.

Mechanistic parallels between sickness and sleep health may help explain the overlapping relationships of sickness and poor sleep health with frustration. A putative pathway by which poor sleep health and sickness, or inflammation, affect emotional functioning is through amplification of amygdala reactivity to emotional stimuli^18,19,40–42^. Frontal cortical regions such as the medial prefrontal cortex can modulate amygdala reactivity, and these areas together are thought to coordinate contextually appropriate emotional responses^18,43^. Individuals exposed to an inflammatory stimulus (i.e., LPS) showed increased amygdala activity in response to socially threatening images^41^ and inflammation in patients with depression predicted decreased amygdala-PFC connectivity^44^. Similarly, it has been shown that 35 h of wakefulness resulted in loss of functional connectivity of the amygdala with areas of the prefrontal cortex (PFC) during exposure to negative images^18^. Decreased amygdala-PFC connectivity after prolonged sleep restriction (i.e., 5 nights of 4 h) was associated with a deterioration of mood in response to negative emotional stimuli, suggesting a neural pathway for sleep-loss induced emotional instability^45^. In the light of neuroimaging research showing that the amygdala-PFC network is likewise implicated in frustration^10^, it is tempting to speculate that stressors such as sickness and poor sleep may impose an additional impact on this network and thereby increase vulnerability to frustration. Research aimed at identifying the neural pathways through which sickness and poor sleep health may affect frustration is not only relevant for everyday life fluctuations in health and sleep, but also for the wide array of psychiatric disorders in which disruption of the amygdala-PFC network is implicated, including mood disorders, anxiety disorder, post-traumatic stress disorder, and personality and conduct disorders^46–48^.

Due to the cross-sectional nature of the current study, causal relationships could not be assessed. Future work is needed in order to establish a causal role of sickness and sleep health in frustration, perhaps via experimentally inducing sickness using lipopolysaccharides (LPS) and manipulations of sleep quality or sleepiness. Addressing whether alleviation of sickness symptoms and sleep restoration can reverse frustration can further provide insight into cause and effect relationships. Lower emotional well-being, due to e.g. stress or depression, has been associated with slower wound healing and recovery from infections and cancer^49–51^. Frustration may likewise negatively impact recovery trajectories of acute or long-term illness, that could thus be potentiated when feeling sick or sleepy, a hypothesis that could be assessed in future research. A further limitation was the inability to control the participants’ surroundings. In order to minimise the influence of this, participants were asked to find a quiet space, turn off their phone, and to remove other potential sources of distraction. Moreover, attention checks and quality checks enabled us to remove those participants who appeared not to have devoted full attention to the study. It has been shown that online studies do seem to produce similar findings to laboratory-based studies, thus providing further validity to the current study^52,53^. A third limitation is the measurement of a single night of sleep and the self-reported nature of sleep duration and stressors. Even though one night of low sleep quality or short sleep duration is common, it is unknown whether participants experienced a single night or chronic low sleep quality or short sleep duration. Whether effects are even larger for those with clinical sleep disturbances remains unknown. Strengths of the study include a pre-registration, the large sample size without stringent exclusion criteria, robust effects that were mostly replicated in an independent sample, and the multifactorial approach to measuring sickness and sleep health, factors that all increase the generalisability and reliability of the results.

In sum, the current study provides evidence for naturally occurring variations in sickness and sleep health being associated both with general frustration and altered responses to a frustrating situation. Contrary to our hypotheses, feeling sick, sleepy, or having low sleep quality was not related to a stronger frustration response when engaged in a frustrating task. Instead, baseline frustration was at higher levels for these individuals. Considering that those with mild sickness or moderate sleep health often do not refrain from participating in the activities of daily life^54^, these results have important implications for many everyday situations. As such, these findings warrant a more comprehensive program of research addressing the causes, consequences, and mechanisms of frustration as related to mild sickness and sleep health (e.g., cortisol levels, heart rate variability, or amygdala reactivity).

## Method

### Participants

Five hundred and seventeen participants (*n* = 265 females, *n* = 230 males, information on sex for 22 participants is missing) (*M* age = 30.4, *SD* = 10.4, range 18–70) were included in the final sample, recruited via an online recruitment platform that facilitates academic research (Prolific.co). Individuals fluent in English and 18 years or older were invited to participate. No other inclusion or exclusion criteria applied. Initially, 504 participants were recruited, of which 52% (*n* = 260) failed to adhere to all the task instructions (see further under ‘4.3.3 Frustration Tolerance Task’). Task instructions were clarified and we continued to enrol participants until valid data was collected for at least 500 participants. In this second round of participant recruitment (*N* = 354) 23% (*n* = 80) were excluded for failing to adhere to the task instructions. To control for possible inattention during the online study, two quality checks and one honesty check were included^55^: “Please rate the response alternative 'agree (9)' for this question”; “Please answer 100”; “Have you been completely honest in your answers?”. Participants who failed more than one quality check (*n* = 1) were excluded before analysis. Table 1 shows the descriptive characteristics of the 517 participants that fulfilled all inclusion criteria and adhered to the task instructions. To assess replicability of results, an additional independent sample of 155 participants was recruited of which 26% (*n* = 40) failed to adhere to the task instructions and an additional two failed the quality check. The remaining sample consisted of 113 participants (*n* = 57 females, *n* = 56 males) (*M* age = 29.0, *SD* = 9.9, range 18–69) (see Supplementary Materials Table S3 for the descriptive characteristics of the study population of this replication study).

The study was conducted according to the guidelines laid down in the Declaration of Helsinki and the protocol was approved by the Swedish Ethical Review Board (2020-03250). Participants provided online informed consent before taking part in the study.

### Procedures

All participants completed the study online. Questionnaires on demographics, mood, sickness, infection-related symptoms, and sleep were completed, followed by the Frustration Tolerance Task based on Feather^56^. Participants received monetary compensation at a rate of £7.50 per hour. The study took about 10 min to complete.

### Measures

#### Sickness, mood, frustration, and self-rated health

An adapted version of the 10-item Sickness Questionnaire was used to assess perceived sickness behaviour^57^. Presence and severity of infection-related sickness symptoms were assessed with eight additional items, and another eight items were used to assess mood, including one item for frustration (baseline frustration). The infection-related sickness items were formulated as ‘I have (a)/I am:’ followed by the item: runny/stuffy nose; loss of smell and/or taste; cough; shortness of breath; sneezing; muscle aches; sore throat; fever. Headache and nausea were included in the Sickness Questionnaire. Frustration and the mood items were formulated as ‘I feel:’ followed by the item: frustrated; lonely; focused; optimistic; anxious; sad; angry; energised. The item ‘I feel depressed’ was already included in the Sickness Questionnaire and was therefore not added as an additional mood item. All items were rated on a 10-point Likert scale where 0 is ‘disagree’, 3 ‘agree somewhat’, 6 ‘mostly agree’, and 9 ‘agree’. Self-rated health (“How would you rate your health at this moment?”) was rated on a 10-point scale from 1 ‘very bad’ to 10 ‘excellent’.

#### Sleepiness, sleep quality, and sleep duration

Sleep health has several dimensions, including sleepiness, sleep quality, and sleep duration^58^. These different dimensions of sleep health have only some overlap. For example, it has been shown that sleep quality is more strongly predicted by stress, health, and waking times than sleep duration (e.g.,^59^) and sleepiness is more of a momentary measure that is not only related to how much or how well we have slept^60^. The Karolinska Sleepiness Scale (KSS) was used to measure current subjective sleepiness, rated on a 9-point scale, ranging from 1 ‘very alert’ to 9 ‘extremely sleepy, fighting sleep, an effort to stay awake’^61^. Subjects reported the previous night’s time of turning the lights out, minutes to falling asleep, and wake time, from which the previous night’s sleep duration was calculated. Subjective sleep quality (rated on a 5-point Likert scale from 1 ‘very bad’ to 5 ‘very good’) was taken from the Karolinska Sleep Diary^62^.

#### Frustration tolerance task

In the Frustration Tolerance Task, based on Feather^56^, participants were presented with eight different geometric shapes, shown below each other on the screen (see Supplementary Materials Fig. S4 for examples of the shapes). Participants were instructed to copy each shape on a piece of paper while adhering to the following rules: the entire shape had to be drawn without lifting the pencil from the paper and without tracing the same line twice. Participants were given three minutes to copy as many of the geometric shapes as possible. A clock displaying the time left for the entire task was visible on the screen. A key component of frustration tolerance is locus of control. When individuals perceive that the environment delays an outcome, they typically become more frustrated^63^. Therefore, in order to induce frustration, unbeknownst to the participants, four out of the eight shapes were unsolvable. Immediately after the task, participants rated how they felt during the task. Visual analogue scales (VAS), ranging from 0 ‘very low / very little’ to 100 ‘very high / very much’, were completed. Participants were instructed to move the marker to a position that reflected how they felt when doing the task, formulated as ‘Move the marker to answer the questions of how you felt when doing the test’, followed by ‘How [*item*] were you?’ with frustration as well as the following mood items: focused; optimistic; anxious; sad; angry; energised; depressed; motivated; ashamed; excited. Another set of VAS were completed to obtain information about the task on mental demand; physical demand; hurriedness; enjoyment; self-perceived performance; amount of effort used; feeling of being in control. Focus, optimism, anxiety, frustration, sadness, anger, energy, and depression ratings were thus obtained at baseline (as part of the sickness and mood ratings, using a 10-point Likert scale) as well as during the task (rated immediately after the task, using a 0–100 VAS in the initial study and using a 10-point Likert scale in the replication study). At the end of the study, participants were asked to enter the number of attempts they made for each of the shapes and whether the shape was successfully completed or not. Participants who reported that they solved two or more of the four unsolvable figures were excluded before analysis. Based on this criterion, 260 out of 504 participants were excluded in the first round of data collection. Therefore, task instructions were clarified by repeating the task rules on the page where the shapes were displayed and data collection resumed until valid data was collected for at least 500 participants. As shown in the Supplementary Materials Table S5, task responses are very similar with and without those who reported having solved none or one unsolved shape. However, importantly, when including all participants (also those who reported two or more times having solved an unsolvable shape) the task did not induce frustration. This further provides support for the fact that the unsolvable nature of the shapes is necessary for inducing frustration.

### Statistical analysis

Data were analysed using SPSS v.24.0 (IBM-SPSS Inc., Chicago, IL, USA) and JASP (Version 0.13 JASP Team 2020). The data and preregistration are available in the Open Science Framework repository (https://osf.io/m9s7a/?view_only=451545ad60ad492bb391cdb7300848f6).

#### Frustration at baseline

A set of mixed effect regression models, including a random effect for the intercept to allow for variation in level between subjects, was estimated for each sickness (depression-like and infection-like sickness, and self-rated health) and sleep health (sleepiness, sleep quality, and sleep duration) predictor for frustration at baseline. Each predictor was tested separately in a univariate model.

#### Sensitivity to becoming frustrated

To assess whether individual differences in sickness and sleep health are associated with the sensitivity to be becoming frustrated, baseline and during the task frustration ratings were subjected to mixed effect models, as per pre-registration. Model simplicity and likelihood ratio tests were used to select appropriate covariance structures. Task (baseline, task) was entered as a repeated and fixed factor and subject was entered as a random factor with a random intercept. The effects of interest were main effects of the predictor and interactions between task and the predictor. Tests of simple main effects were performed on the linearly independent pairwise comparisons between the estimated marginal means. All results of the regression models are presented as standardised coefficient estimates (β). The main focus of the analysis was prediction of the change in frustration from baseline to during the task from sickness and sleep health.

Before analysis, baseline frustration values were rescaled to the lower and upper limits of the frustration rating during the task, measured using a 0–100 VAS. The original value (rated on a 10-point scale) was divided by the range of the original scale (9) multiplied by the upper limit of the rescaled variable (100)^64^. To assess replicability of results and to rule out the possibility that the use of different scales for baseline (0–9 Likert scales) and during-task (0–100 VAS) ratings affected the results, the study was repeated using identical study procedures except for the during-task ratings which used the same 0–9 Likert scale as was used for the baseline ratings. The during-task items asked how participants felt during the task, formulated as ‘I felt:’ followed by the item.

### Exploratory analyses

#### Different aspects of sickness

In the preregistration we described the aim to assess the relationships between frustration and three sickness measures: overall sickness score obtained using the Sickness Questionnaire, flu-like symptoms, and self-rated health. However, to determine whether the sickness variables of the Sickness Questionnaire and the flu-like symptoms indeed capture different aspects of sickness, exploratory factor analysis was performed. For this purpose, all sickness symptoms (Sickness Questionnaire items and flu-like symptoms), and all mood items, except for the dependent variable frustration, were entered and components with eigenvalues above 1.0 were extracted using oblique promax rotation. Maximum likelihood was used as the estimation method. The scree plot and a primary factor loading of > 0.3 was used to identify the factors. The identified factors were then used to compute composite scores by calculating the sum of the item ratings for each factor. Positive items were reverse scored before creating composite scores (i.e., energised, focused, optimistic). These scores were used to predict frustration at baseline and sensitivity to becoming frustrated (i.e., task-induced frustration). The results obtained using the newly identified factors are almost identical to the results obtained with overall sickness as predictor of frustration. However, since the component analysis is a slight deviation from the pre-registration, the results with overall sickness are shown in the Supplementary Material ‘5.4 Sickness Questionnaire’.

#### U-shaped relationship between sleep duration and frustration

U-shaped relationships are often observed between sleep duration and health outcomes^65,66^. To test whether a similar U-shaped relationship exists between sleep duration and frustration, a curve estimation regression model was run. A linear model was used, then a quadratic term was added and models were compared.

#### Different responses for those low, intermediate or high in each stressor

Individuals were divided into groups based on their baseline stressor level in order to assess whether frustration at baseline or the sensitivity to becoming frustrated differed as a function of stressor level. We used cut-off scores for sleep duration, sleep quality, and self-rated health, and quartiles (25%, 50%, 25%) for sleepiness and for the sickness factors, in order to divide the sample into groups of low, intermediate, and high baseline levels of that particular stressor*.* Short sleep duration was defined as < 7 h (the minimum recommended sleep duration for adults to maintain health; ^67^), normal sleep duration as ≥ 7 h to < 9 h, and long sleep duration as ≥ 9 h. Poor sleep quality was defined as a score of 1 or 2, intermediate sleep quality as 3, and good sleep quality as 4 or 5. Poor self-rated health were those with a rating of 5 or lower, intermediate self-rated health were those with a rating of 6 or 7, and good self-rated health was defined as 8 or higher. Baseline and during-task frustration ratings were subjected to mixed effect models, in a similar fashion as was pre-registered for the analysis with the predictors as continuous variables. Task was entered as a repeated and fixed factor, and subject was entered as a random factor with a random intercept. The effects of interest were interactions between task and group.

## Supplementary Information

Supplementary Information

## Data Availability

The dataset and preregistration are available in the Open Science Framework repository (https://osf.io/m9s7a/?view_only=451545ad60ad492bb391cdb7300848f6).
