# Supplementary material for: Sickness and sleep health predict frustration and affective responses to a frustrating trigger
Source: Sci Rep. 2021 Jan 15;11:1542. doi: 10.1038/s41598-020-80461-4 (PMC7810868; doi:10.1038/s41598-020-80461-4)
Supplement: Supplementary file 1 — Supplementary Information [file 41598_2020_80461_MOESM1_ESM.pdf]

SUPPLEMENTARY MATERIAL

Sickness and Sleep Health Predict Frustration and Affective Responses to a Frustrating Trigger

Leonie JT Balter[1*], Tina Sundelin[1,2], & John Axelsson[1,2]
[1]Stress Research Institute, Stockholm University, Stockholm, Sweden
[2]Department of Clinical Neuroscience, Karolinska Institutet, Solna, Sweden

* Corresponding author: Leonie Balter, leonie.balter@su.se
Tina Sundelin: tina.sundelin@ki.se
John Axelsson: john.axelsson@su.se

5.1 Figures

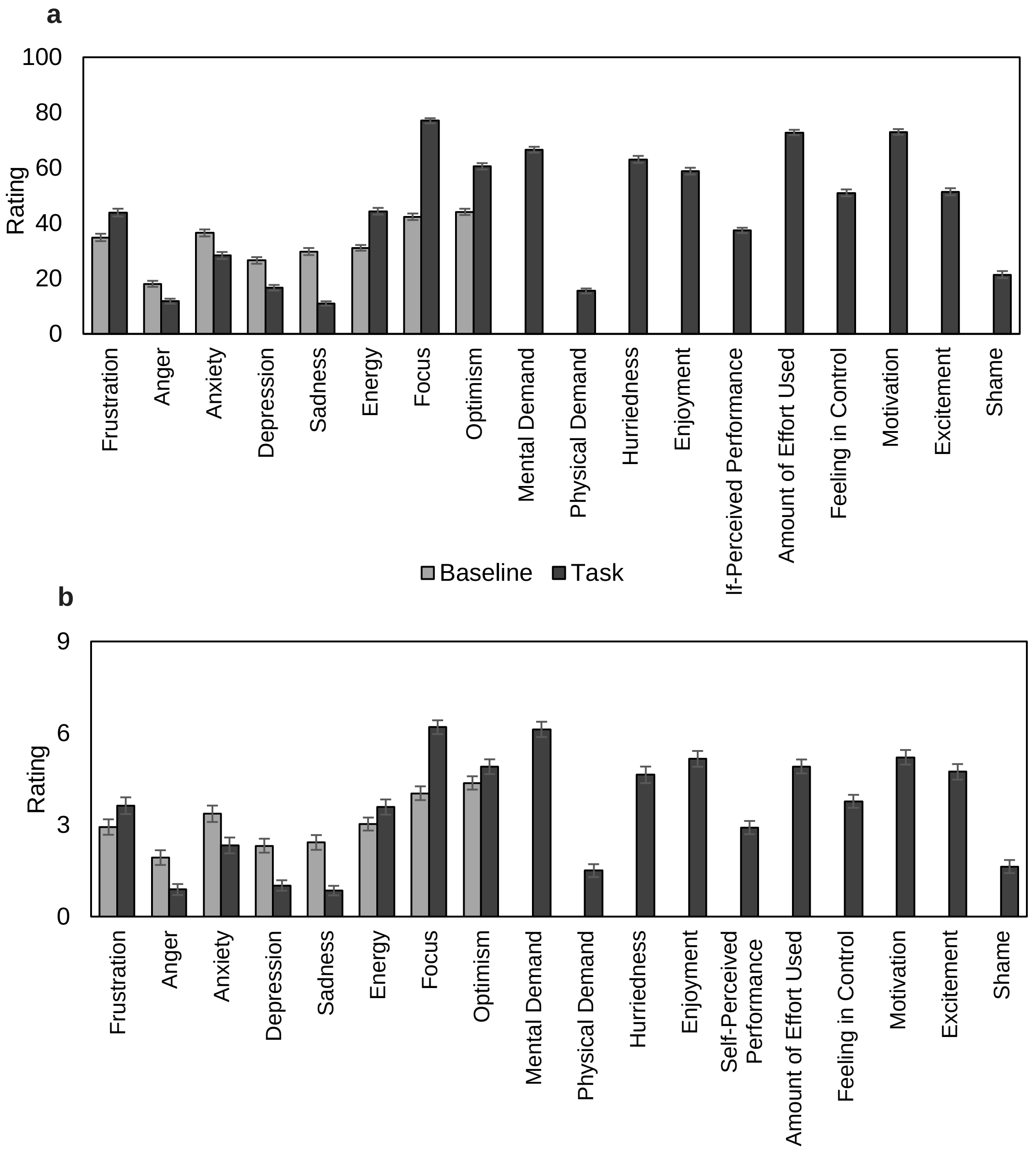

Figure S1. Replication of the task responses in independent samples. Mean (± SEM) ratings at baseline and during the Frustration Tolerance Task (the during-task ratings concerned their feeling during the task, rated immediately after the task) for (a) *N* = 517, with baseline ratings rescaled to the same during-task 0-100 VAS, and (b) *N* = 113, with baseline and during-task ratings measured on a 10-point (0-9) Likert scale.

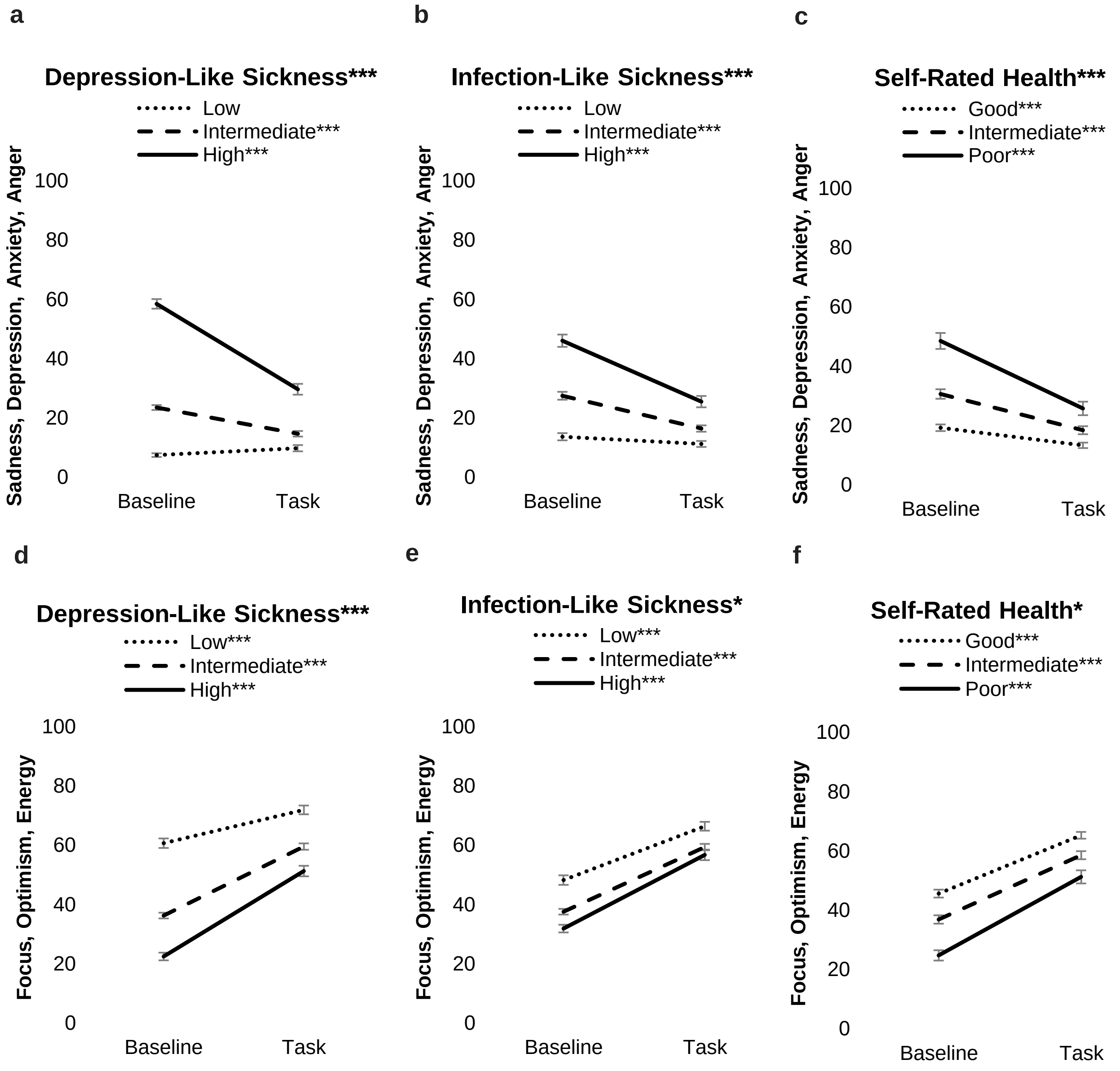

Figure S2. Negative (a, b, c) and positive (d, e, f) mood (means ± SEM) at baseline and during the Frustration Tolerance Task (the during-task ratings concerned their feeling during the task, rated immediately after the task) separated by sickness groups (N = 517). Y-axes show the average rating of the mood items (0-100). Higher scores indicate greater negative (a, b, c) or greater positive (d, e, f) mood. Significant interaction effects are indicated with asterisks in the title. Asterisks in the legends indicate significance level for post-hoc task effects (baseline, task) in case a significant stressor x task interaction was evident. $^{\#}$ $p < .07$, * $p < .05$, ** $p < .01$, *** $p < .001$.

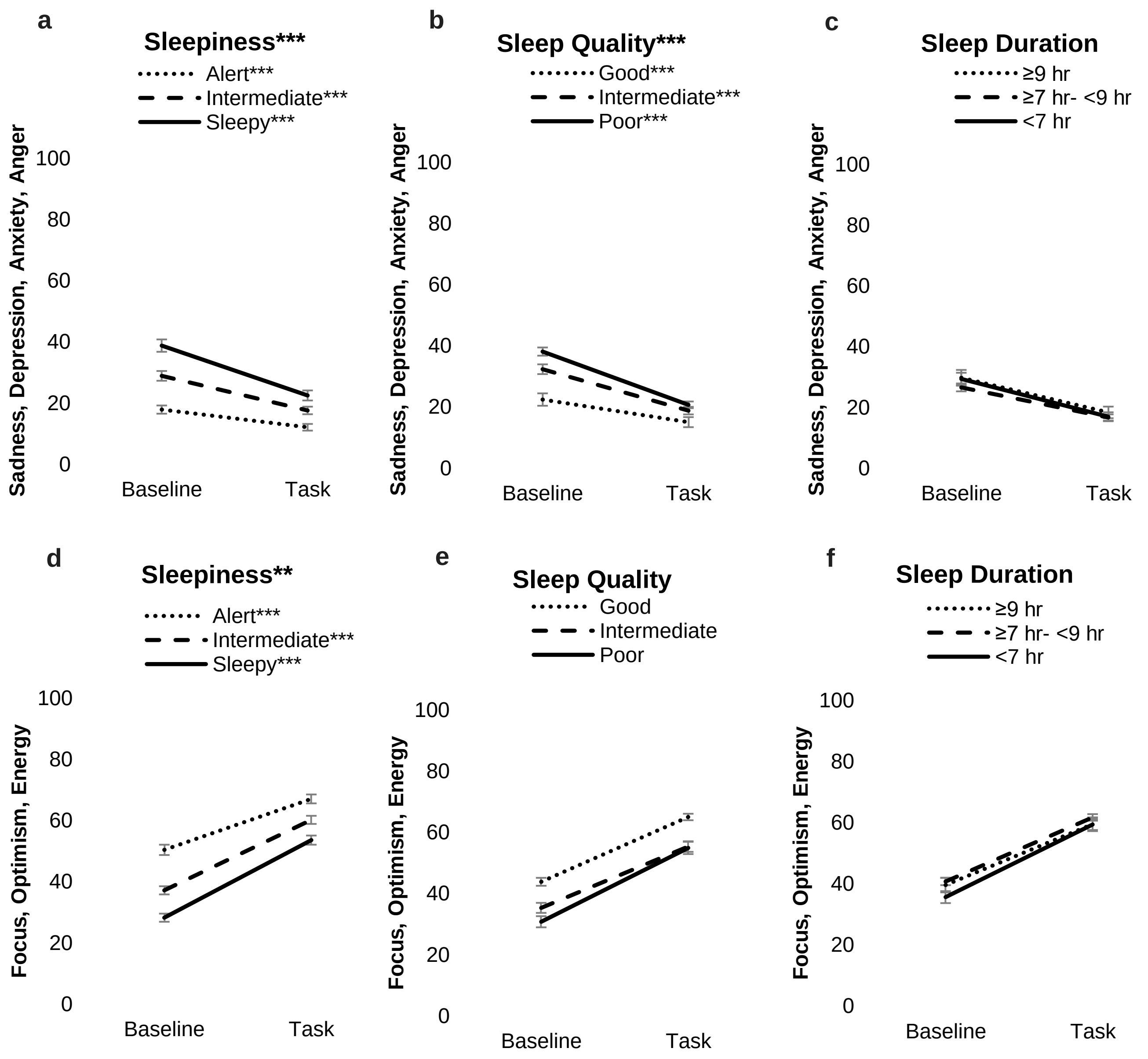

Figure S3. Negative (a, b, c) and positive (d, e, f) mood (means ± SEM) at baseline and during the Frustration Tolerance Task (the during-task ratings concerned their feeling during the task, rated immediately after the task) separated by sleep health groups (*N* = 517). Y-axes show the average rating of the mood items (0-100). Higher scores indicate greater negative (a, b, c) or greater positive (d, e, f) mood. Significant interaction effects are indicated with asterisks in the title. Asterisks in the legends indicate significance level for post-hoc task effects (baseline, task) in case a significant stressor x task interaction was evident. $^{\#}$ $p < .07$, * $p < .05$, ** $p < .01$, *** $p < .001$.

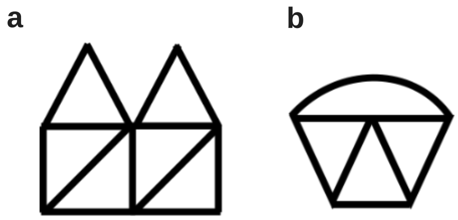

Figure S4. Example of a solvable (a) and an unsolvable (b) geometric shape.

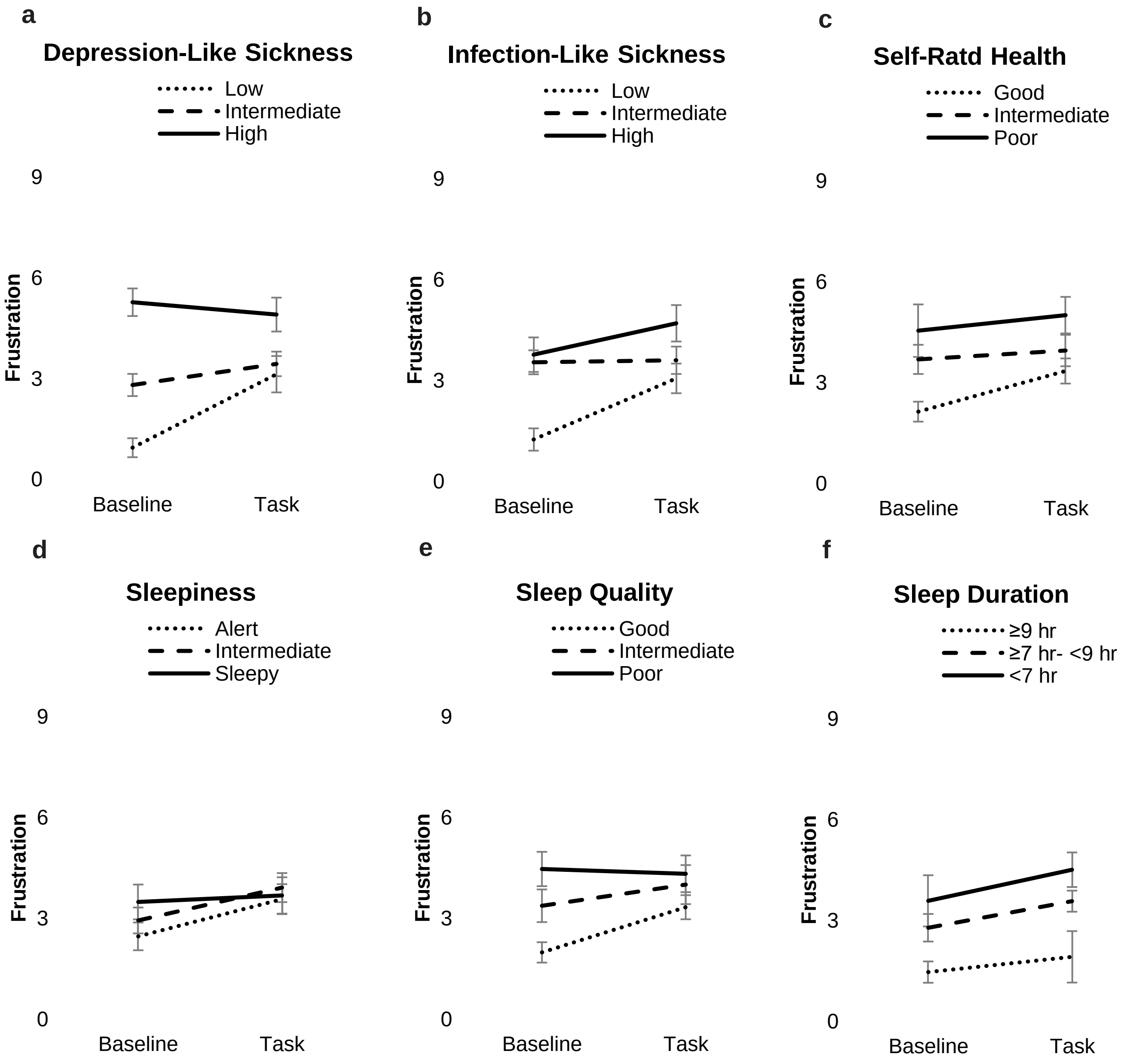

Figure S5. Frustration ratings (means ± SEM) of the replication study, at baseline and during the Frustration Tolerance Task (rated post task) separated by sickness groups (a, b, c) and sleep health groups (d, e, f). Higher scores indicate greater frustration.

5.2 Tables

Table S1. Factor loadings based on exploratory component analysis with promax rotation for the 25 sickness-related symptoms. The item “I want to keep still” was eliminated because the item failed to meet the criterium of having a factor loading of >0.3 and did not load onto any of the two factors. [1] = items used to calculate the composite Factor 1 score, [2] = items used to calculate the composite Factor 2; Factor 1 and Factor 2 explained 29.2% and 9.9% of the variance, respectively.

	Factor 1	Factor 2
Depressed	0.798[1]	0.065
Sad	0.714[1]	0.099
Drained	0.751[1]	0.110
Optimistic	-0.722[1]	0.213
Tired	0.632[1]	0.123
Angry	0.534[1]	0.155
Anxious	0.531[1]	0.187
Lonely	0.535[1]	0.057
Energised	-0.557[1]	0.125
Focused	-0.527[1]	0.118
Do not wish to do anything	0.531[1]	-0.043
Wish to be alone	0.385[1]	0.008
Shortness of breath	-0.108	0.711[2]
Cough	-0.167	0.654[2]
Sore throat	-0.160	0.650[2]
Fever	-0.162	0.614[2]
Headache	0.200	0.523[2]
Muscle aches	0.096	0.510[2]
Sneezing	-0.023	0.501[2]
Nauseous	0.207	0.478[2]
Runny/stuffy nose	-0.017	0.443[2]
Shaky	0.225	0.424[2]
My body feels sore	0.258	0.337[2]
Loss of smell/taste	0.098	0.314[2]
Want to keep still	0.192	-0.040

Table S2. Original (before rescaling) mean frustration ratings (SEM) at baseline rated on a 10-point scale (0-9), separated by low/short, intermediate, and high/long stressor groups. Please note that lower depression-like sickness, infection-like sickness, and sleepiness indicate a better level of the stressor, while lower sleep quality and self-rated health indicate a worse level of the stressor. Short sleep duration refers to <7 hr, intermediate sleep duration to ≥7 to <9 hr, and long sleep duration to ≥9 hr.

	Low/short	Intermediate	High/Long
	Baseline	Baseline	Baseline
Depression-like sickness	1.13 (0.08)	2.92 (0.13)	5.74 (0.25)
Infection-like sickness	1.55 (0.10)	3.16 (0.15)	5.06 (0.23)
Self-rated health	5.24 (0.19)	3.31 (0.13)	2.34 (0.19)
Sleep quality	4.11 (0.21)	3.47 (0.18)	2.67 (0.14)
Sleepiness	2.22 (0.13)	3.30 (0.17)	4.06 (0.19)
Sleep duration	3.02 (0.16)	3.19 (0.15)	3.13 (0.17)

Table S3. Descriptive characteristics of the study population of the replication study given as sample size (*n*) and percentage (%) and means (*M*) and standard deviation (*SD*). Percentages may not total 100 due to rounding.

Variable	*n* (%)	*M* (*SD*)
N	113	
Age (years)		29.0 (9.9)
Sex		
Male	57 (50%)	
Female	56 (50%)	
Depression-like sickness (range 4 – 93)		41.2 (20.8)
Low	32 (28%)	17.9 (7.0)
Intermediate	51 (45%)	39.4 (7.5)
High	30 (27%)	69.1 (10.2)
Infection-like sickness (range 0 – 77)		12.1 (13.8)
Low	33 (29%)	1.3 (1.1)
Intermediate	50 (44%)	8.4 (3.4)
High	30 (27%)	30.3 (14.5)
Self-rated health *M* (*SD*) (range 3 – 10)		7.5 (1.6)
Low (<6)	13 (12%)	4.6 (0.6)
Intermediate (6 or 7)	38 (34%)	6.6 (0.5)
High (8, 9, or 10)	62 (55%)	8.7 (0.7)
Sleepiness (range 1 – 9)		4.9 (1.8)
Low	37 (33%)	2.7 (0.6)
Intermediate	45 (40%)	5.2 (0.6)
High	31 (27%)	7.1 (0.4)
Sleep quality (range 1 – 5)		3.3 (1.0)
Low (1 or 2)	28 (25%)	1.9 (0.3)
Intermediate (3)	27 (24%)	3.0 (0.0)
High (4 or 5)	58 (51%)	4.1 (0.3)
Sleep duration (range 3 hr – 17 hr 30)		7.8 (1.5)
Short (<7 hr)	40 (35%) of which *n* = 17 <6 hr	5 hr 54 min (1 hr 03 min)
Normal (≥7–<9 hr)	62 (55%)	8 hr 01 min (0 hr 31 min)
Long (≥9 hr)	11 (10%)	9 hr 38 min (0 hr 25 min)

Table S4. Replication of the baseline and during-task responses in an independent sample (*N* = 113). Mean ratings (SEM) at baseline and during the Frustration Tolerance Task (the 'task' rating concerned their feeling during the task, rated immediately after the task) rated on 10-point Likert scales from 0 to 9. Higher scores indicate a greater level of the variable (e.g., higher anxiety, higher energy, greater effort used).

	Baseline	Task	*β*	95% CI	*p*
Frustration	2.9 (0.3)	3.7 (0.3)	0.28	0.06, 0.51	.013
Anger	1.9 (0.2)	0.9 (0.2)	-0.43	-0.64, -0.23	<.001
Anxiety	3.4 (0.3)	2.3 (0.3)	-0.36	-0.57, -0.15	.001
Depression	2.3 (0.2)	1.0 (0.2)	-0.57	-0.73, -0.41	<.001
Sadness	2.4 (0.2)	0.9 (0.2)	-0.66	-0.88, -0.46	<.001
Energy	3.0 (0.2)	3.5 (0.2)	0.21	0.00, 0.42	.050
Focus	4.0 (0.2)	6.2 (0.2)	0.82	0.62, 1.03	<.001
Optimism	4.4 (0.2)	4.9 (0.2)	0.21	0.02, 0.41	.030
Mental demand		6.2 (0.2)			
Physical demand		1.5 (0.2)			
Hurriedness		4.7 (0.3)			
Enjoyment		5.1 (0.3)			
Self-perceived performance		2.9 (0.2)			
Amount of effort used		5.0 (0.2)			
Feeling in control		3.7 (0.2)			
Motivation		5.2 (0.2)			
Excitement		4.7 (0.3)			
Shame		1.6 (0.2)			

Table S5. Mean ratings (SEM) at baseline and during the Frustration Tolerance Task (the ‘task’ rating concerned their feeling during the task, rated immediately after the task), shown separately for ‘All participants’ (n = 858), ‘Max 1 unsolvable solved’ (those who reported having solved an unsolvable shape none or one time; n = 517), and ‘0 unsolvable solved’ (those who reported all four unsolvable shapes as unsolved; n = 382). Higher scores indicate a greater level of the variable (e.g., higher frustration, higher anger, higher energy).

Frustration and other mood items	Exclusion criteria	Baseline	Task
Frustration	All participants	35.4 (1.0)	34.7 (1.1)
	Max 1 unsolvable solved	34.9 (1.3)	43.9 (1.4)
	0 unsolvable solved	35.3 (1.6)	45.6 (1.7)
Anger	All participants	19.7 (0.9)	11.1 (0.7)
	Max 1 unsolvable solved	18.1 (1.1)	11.8 (0.9)
	0 unsolvable solved	17.4 (1.2)	12.3 (1.1)
Anxiety	All participants	37.1 (1.0)	25.9 (1.0)
	Max 1 unsolvable solved	36.5 (1.3)	28.3 (1.3)
	0 unsolvable solved	36.4 (1.4)	29.0 (1.5)
Depression	All participants	26.8 (0.9)	15.9 (0.8)
	Max 1 unsolvable solved	26.5 (1.2)	16.7 (1.0)
	0 unsolvable solved	26.3 (1.4)	17.3 (1.3)
Sadness	All participants	31.0 (1.0)	11.5 (0.7)
	Max 1 unsolvable solved	29.8 (1.3)	11.0 (0.8)
	0 unsolvable solved	30.1 (1.5)	11.4 (1.0)
Energy	All participants	32.6 (0.9)	45.4 (1.0)
	Max 1 unsolvable solved	31.1 (1.0)	44.3 (1.2)
	0 unsolvable solved	30.1 (1.2)	43.6 (1.4)
Focus	All participants	42.3 (0.9)	76.9 (0.8)
	Max 1 unsolvable solved	42.3 (1.2)	77.0 (0.9)
	0 unsolvable solved	41.9 (1.4)	77.5 (1.1)
Optimism	All participants	44.9 (0.9)	63.2 (0.9)
	Max 1 unsolvable solved	44.1 (1.1)	60.6 (1.2)
	0 unsolvable solved	43.8 (1.3)	59.4 (1.4)

5.3 Results of the replication study

5.3.1 Sample characteristics

Sample characteristics of the study population of the replication study are summarised in Table S3.

5.3.2 Replication of task-induced frustration and mood

As shown in Figure S1 and Table S4, all task responses were replicated in an independent sample of participants, indicating strong replicability of task responses and that the 10-point Likert scale can be validly rescaled to the 0-100 VAS.

5.3.3 Replication of baseline frustration and stressors

Depression-like sickness, infection-like sickness, self-rated health

All three sickness-related factors predicted frustration at baseline: depression-like sickness (β = 0.63, 95% CI [0.49, 0.78], p < .001), infection-like sickness (β = 0.34, 95% CI [0.17, 0.52], p < .001), and self-rated health (β = -0.33, 95% CI [-0.51, -0.16], p < .001). However, the small variation in self-rated health in this sample (there were only 13 (12%) participants who rated their health as low) limits the validity of the sleep duration analysis.

Sleepiness, sleep quality, and sleep duration

Lower sleep quality (β = -0.43, 95% [CI -0.60, -0.26], p < .001) predicted higher frustration at baseline. Although in the same direction as in the original study, sleepiness did not significantly predict frustration at baseline (β = 0.14, 95% CI [-0.05, 0.32], p = .142). Sleep duration, however, significantly predicted baseline frustration (β = -0.24, 95% CI [-0.42, -0.06], p = .010). The small variation in sleep duration in this sample (there were only 11 (10%) long sleepers) limits the validity of the sleep duration analysis.

5.3.4 Replication of task-induced frustration

Sickness and sleep health

Figure S5 shows the baseline and during-task frustration ratings separated by individuals low, intermediate, or high in each of the sickness (a, b, c), and sleep health (d, e, f) stressors. Results are mostly in the same direction as in the original study. However, due to the smaller sample size and thus lower variation in sickness and sleep health, we did not have enough statistical power to reach statistical significance for all interaction effects.

5.4 Sickness Questionnaire

On average, the sickness score of the adapted Sickness Questionnaire was 24.1 (*SD* = 14.9; range 0–75), calculated as the sum of all the 10 items rated on 10-point scales from 0 to 9. Converting the scale to the original 4-point scales (0 to 3) showed an average score of 7.5 (*SD* = 4.4, range 0–19).

5.4.1 Sickness Questionnaire and baseline frustration

Sickness, similar to depression-like sickness, infection-like sickness, and self-rated health, predicted frustration at baseline (β = 0.53, 95% CI [0.46, 0.61], p < .001). Those with high sickness reported highest frustration at baseline (M = 5.10, SE = 0.23), followed by those intermediate (M = 2.97, SE = 0.14) in sickness, and lowest frustration levels were observed in those with low sickness (M = 1.55, SE = 0.10) (frustration was rated on a 0-9 Likert scale) (F(2, 517) = 75.17, p < .001).

5.4.2 Sickness Questionnaire and task-induced frustration

A significant task (baseline, task) x sickness interaction (β = -0.28, 95% CI [-0.39, -0.19], p < .001) was evident. Separating sickness into low, intermediate, and high levels showed that those low (*Mdiff* = +17.70, 95% CI [11.32, 23.34], p < .001) and intermediate (*Mdiff* = +11.39,

95% CI [6.88, 15.91], $p < .001$) in sickness increased in frustration while frustration levels in those high in sickness did not significantly change (*M*diff = -4.48, 95% CI [-10.43, 1.90], $p = .175$) (sickness x task; $F(2, 517) = 13.19$, $p < .001$). Before analysis, the baseline ratings, rated on 10-point Likert scales, were rescaled to the 0-100 VAS.
